# COX-2 expression is associated with an aggressive phenotype in ductal carcinoma *in situ*

**DOI:** 10.1038/sj.bjc.6601534

**Published:** 2004-01-20

**Authors:** G P Boland, I S Butt, R Prasad, W F Knox, N J Bundred

**Affiliations:** 1 Academic Department of Surgery, University Hospital of South Manchester, Southmoor Road, Manchester M23 9LT, UK; 2 Department of Pathology, University Hospital of South Manchester, Southmoor Road, Manchester M23 9LT, UK

**Keywords:** DCIS, Breast, COX-2, HER-2, oestrogen, proliferation

## Abstract

Cyclooxygenase type-2 (COX-2) is overexpressed in malignant tumours including breast cancers, though the mechanism of upregulation is unclear. This study aimed to determine COX-2 expression in ductal carcinoma *in situ* (DCIS) in comparison to invasive breast cancer (IBC) and normal breast, and also to investigate the relationship of COX-2 expression with HER-2 expression, oestrogen receptor (ER), tumour grade and cellular proliferation (Ki67) in DCIS. Cyclooxygenase type-2, HER-2, ER and Ki67 expression were determined by immunohistochemistry on paraffin tissue sections of DCIS (*n*=187), IBC (*n*=65) and normal breast reduction tissue (*n*=60). Cyclooxygenase type-2 expression in DCIS (67%, *P*<0.001) and IBC (63%, *P*<0.001) was significantly greater than in normal breast (23%). There was no difference in COX-2 expression level between DCIS and IBC (*P*=0.87) or between normal breast from reduction mammoplasty tissue and normal breast ducts around DCIS (22%, *P*=0.29). In DCIS, COX-2 expression was associated with higher cellular proliferation rates (*P*<0.0001), nuclear grade (*P*=0.003), with ER negativity (*P*=0.003) and with HER-2 positivity (*P*<0.0001). Cyclooxygenase type-2 expression is upregulated in *in situ* breast cancer and is associated with surrogate markers of an aggressive DCIS phenotype including nonoestrogen-regulated signalling pathways. Cyclooxygenase type-2 inhibition may potentially prevent the development of ER-positive and ER-negative breast cancers.

Cyclooxygenase type-2 (COX-2) is overexpressed in many human malignant tumours (Sano *et al*, 1995; Wolff *et al*, 1998; Mohammed *et al*, 1999; Tucker *et al*, 1999; Soslow *et al*, 2000) and has been linked to the process of carcinogenesis (Liu *et al*, 2001), tumour survival (Tsujii *et al*, 1998), invasion (Tsujii *et al*, 1997) and metastasis (Tsujii and DuBois, 1995; Costa *et al*, 2002). Epidemiological studies have reported a significant reduction in the incidence of human gastro-intestinal cancers with COX inhibition by NSAIDS (Shaheen *et al*, 2002). Although the evidence for breast cancer prevention is less strong, a recent meta-analysis of 14 studies (six cohort, eight case-controlled) gave a combined estimate of a reduced relative risk of 0.82 (95% confidence interval 0.75–0.89) with regular NSAID use (Khuder and Mutgi, 2001), but this provided no data on the dose–response effect for NSAID type or duration of use.

Pharmacological studies with selective COX-2 inhibitors in animal models of breast cancer (and other cancers) have consistently demonstrated a dose-dependent arrest of tumour growth, invasion and metastasis (Alshafie *et al*, 2000; Harris *et al*, 2000; Rozic *et al*, 2001; Kundu and Fulton, 2002). These epidemiological and animal data indicate the potential of long-term COX-2 inhibition by the safer new generation of COX-2-specific NSAIDS for breast cancer chemoprevention.

Early studies of COX-2 RNA/protein expression in invasive breast cancer (IBC) yielded inconsistent findings, with expression reported to be between 0 and 100% of samples (Parrett *et al*, 1997; Hwang *et al*, 1998). Immunohistochemical studies of COX-2 antigen expression in IBC have produced more consistent findings, with moderate or strong levels of COX-2 expression found in 36–56% of IBCs (Soslow *et al*, 2000; Half *et al*, 2002; Ristimaki *et al*, 2002; Denkert *et al*, 2003; Spizzo *et al*, 2003; Watanabe *et al*, 2003), with COX-2 expression predicting poorer disease-free survival (Ristimaki *et al*, 2002; Denkert *et al*, 2003; Spizzo *et al*, 2003).

COX-2 upregulation appears to occur early in the process of carcinogenesis, with overexpression reported in a number of premalignant lesions and in *in situ* neoplasia of nonbreast glandular epithelium (Eberhart *et al*, 1994; Kirschenbaum *et al*, 2000; Shirahama, 2000; Morris *et al*, 2001). To date, there are few reports of COX-2 expression in ductal carcinoma *in situ* (DCIS) of the breast. These have been limited to DCIS surrounding invasive cancer (Soslow *et al*, 2000; Half *et al*, 2002), with only two studies in primary DCIS (Shim *et al*, 2003; Watanabe *et al*, 2003). All these studies have looked at less than 50 DCIS tumours, and have shown moderate or strong COX-2 antigen expression in 60–85% of lesions. Shim *et al* (2003) reported a correlation of COX-2 expression and nuclear grade in pure DCIS, although the study lacked power to demonstrate an association with other markers (Shim *et al*, 2003). No single study has yet specifically determined COX-2 expression in pure DCIS compared to invasive cancer and normal breast epithelium (from breast reduction tissue) and the relationship to surrogate molecular markers of pathways driving cell proliferation in DCIS.

Despite the data on elevated COX-2 expression in breast neoplasia, the mechanism of upregulation remains unclear. In this study, we evaluated COX-2 expression in DCIS using immunohistochemistry in comparison to expression in invasive cancer and normal breast, and investigated the association between COX-2 expression and cellular proliferation, nuclear grade and HER-2 antigen expression in DCIS, since HER-2 is overexpressed in at least two-thirds of *in situ* breast cancer (Allred *et al*, 1993; Bobrow *et al*, 1995; Suo *et al*, 2001).

## PATIENTS AND METHODS

### Tissue specimens and selection

A retrospective study was performed on archival paraffin-embedded formalin-fixed tissue samples of normal breast (reduction mammoplasty, *n*=60), DCIS (*n*=187) and IBC (*n*=65) from women who had undergone surgery at the University Hospital of South Manchester. Antigen expression was assessed by immunohistochemistry. Samples were deliberately selected to include a higher proportion of HER-2-positive invasive cancer and a lower proportion of HER-2-negative DCIS than expected by random selection, to compare COX-2 expression in HER-2-expressing/nonexpressing tumours.

### Immunohistochemistry (Figure 3)

An immunohistochemical assay of COX-2, Ki67, oestrogen receptor (ER) and HER-2 was performed on paraffin wax sections (3–5 *μ*m thick) of each tissue were mounted on APES (3-aminopropyltriethoxysilane, Sigma) coated slides, de-waxed in xylene and rehydrated prior to immunohistochemical staining. Established protocols developed at the clinical research laboratory, Paterson Institute for Cancer Research, Manchester (a UK reference laboratory for HER-2 immunohistochemical staining) were followed for each antigen (except COX-2); these have been shown to produce reproducible immunostaining in DCIS (Gandhi *et al*, 2000; Dowsett *et al*, 2001). For all molecular markers, antigen retrieval was achieved by the pressure cooking method for 4 min in citrate buffer (pH=6.0).

For COX-2 expression, a primary goat polyclonal anti-human COX-2 antibody was used (sc-1745; Santa Cruz Biotechnology, USA) as described in other studies assessing COX-2 expression by immunohistochemistry (Komhoff *et al*, 2000; Shirahama, 2000; Cianchi *et al*, 2001; Sales *et al*, 2001; Shirahama *et al*, 2001; Hasturk *et al*, 2002; Muller-Decker *et al*, 2002) at a dilution of 1 : 100 for 1 h, followed by a biotinylated rabbit anti-goat secondary antibody (DAKO, Z259) diluted 1 : 200 for 40 min. Cyclooxygenase type-2 immunoreactivity was confirmed with another COX-2 primary antibody (Cayman Chemical Company, Ann Arbor, MI, USA, 160112) in 50 DCIS sections. For Ki67 (MIB-1), a primary mouse monoclonal antibody to MIB-1 was used (DAKO Ltd, UK, M7240) at 1 : 50 for 1 h, followed by a biotinylated goat anti-mouse secondary antibody (DAKO Ltd, UK, E432) diluted 1 : 200 for 40 min. For ER, a primary mouse anti-human ER antibody was used (DAKO Ltd, UK, M7047), at 1 : 33 for 1 h, followed by a biotinylated secondary goat anti-mouse antibody (DAKO Ltd, UK, E432), at 1 : 200 for 40 min. For HER-2 labelling, a primary mouse anti-human HER-2 antibody was used (DAKO Ltd, UK, A485) at 1 : 40 for 1 h, followed by a biotinylated secondary rabbit anti-mouse antibody (DAKO Ltd, UK, E413), at a 1 : 200 for 40 min. Antigen visualisation was achieved by applying a standard streptavidin–biotin complex (ABC, Vector labs, UK, PK-6100) for 30 min followed by diaminobenzidene chromogen (DAB, DAKO, UK) in 0.1% H_2_O_2_ PBS solution. Sections were counterstained with Gill's haematoxylin.

A positive and negative control slide was included in each immunohistochemical assay. The positive controls used were as follows: COX-2 expression: high-grade transitional carcinoma of the urinary bladder and skin (Mohammed *et al*, 1999; Komhoff *et al*, 2000; Ristimaki *et al*, 2001); HER-2 expression: strongly HER-2-positive (3+) IBC; ER, PR and Ki67 expression: DCIS known to express the appropriate antigen as determined in previous studies of DCIS in our department. For negative controls, the appropriate primary antibody was omitted and either PBS or an iso-type matched IgG serum was applied. A specific COX-2 blocking peptide (Cayman Chemical Company, Ann Arbor, MI, USA, 360107) was used as a negative control in 50 DCIS sections stained with the Cayman anti-COX-2 antibody (160112) as previously described (Half *et al*, 2002).

### Evaluation of immunostaining

Immunostaining was evaluated by light microscopy blindly and independently by GPB and ISB, and a consensus agreement was achieved. Cyclooxygenase type-2 expression was scored 0 (absent), 1+ (weak), 2+ (moderate) and 3+ (strong) based on the extent and intensity of epithelial cell staining (Half *et al*, 2002; Ristimaki *et al*, 2002; Shim *et al*, 2003). Cyclooxygenase type-2 positivity was defined as a score ⩾2 (Shamma *et al*, 2000; Ristimaki *et al*, 2002; Shim *et al*, 2003). HER-2 staining was scored 0 (absent) to 3 (maximum cyto-membranous staining seen, comparable to a 3+ positive invasive cancer control), with a score ⩾2 considered HER-2 positive (Birner *et al*, 2001). Ki67 and ER scores were calculated as the percentage of positively stained nuclei. Oestrogen receptor positivity was defined as ⩾5% stained nuclei, and has been used in previous studies in DCIS in our unit (Holland *et al*, 1997; Gandhi *et al*, 1998,^2000^; Boland *et al*, 2003b). For each section, a minimum of 1000 cells were scored across randomly selected areas of DCIS at a magnification of × 400 using a grid graticule and cell counter.

### Statistical analysis

Cyclooxygenase type-2 expression scores between different breast tissues were compared using the Mann–Whitney test. The relationship between categorical variables was analysed using the *χ*^2^ test and the association of categorical variables with continuous variables analysed using the Kruskal–Wallis and Mann–Whitney tests. Significance tests were two-tailed and a significance level of 5% was used throughout.

## RESULTS

### COX-2 immunostaining in different breast tissues

Cyclooxygenase type-2 immunostaining was determined in 372 samples of normal and neoplastic breast ductal epithelium (Table 1

**Table 1:** COX-2 expression in normal and neoplastic breast

		**COX-2 score (0=absent to 3=maximum)**					
**Tissue**	**No.**	**0**	**1**	**2**	**3**	**No. COX-2 +ve (score ⩾2+)**	***P*-value^b^**
NB	60	25 (42%)	21 (35%)	12 (20%)	2 (3%)	14/60 (23%)	Reference
NB around DCIS	60	33 (55%)	14 (23%)	9 (15%)	4 (7%)	13/60 (22%)	0.29
*DCIS*							
Low^a^	27	4 (15%)	11 (41%)	6 (22%)	6 (22%)	12/27 (44%)	
Intermediate^a^	64	8 (13%)	15 (23%)	27 (42%)	14 (22%)	41/64 (64%)	
High^a^	96	8 (8.3%)	16 (17%)	37 (39%)	35 (36%)	72/96 (75%)	
Total	187	20 (11%)	42 (23%)	70 (37%)	55 (29%)	125/187 (67%)	<0.0001

*Invasive cancer*							
Grade 1	17	2 (12%)	6 (35%)	2 (15%)	7 (41%)	9/17 (53%)	
Grade 2	17	1 (6%)	6 (35%)	6 (35%)	4 (45%)	10/17 (58%)	
Grade 3	31	3 (10%)	6 (19%)	8 (26%)	14 (45%	22/31 (71%)	
Total	65	6 (9%)	18 (28%)	16 (25%)	25 (39%)	41/65 (63%)	<0.0001

NB was from breast reduction tissue and ducts surrounding DCIS. Cyclooxygenase type-2 expression was scored 0 (absent) to 3 (maximum intensity seen) on tissue sections from each patient, with a score ⩾2 considered COX-2 expression positive. The *P-*values (Mann–Whitney test) are for comparison to COX-2 expression in NB from breast reduction tissue (Referent). COX-2=cyclooxygenase type-2; NB=normal breast; DCIS=ductal carcinoma *in situ*.

a DCIS nuclear grade.

b Mann–Whitney test.

, Figure 1

**Figure 1:**
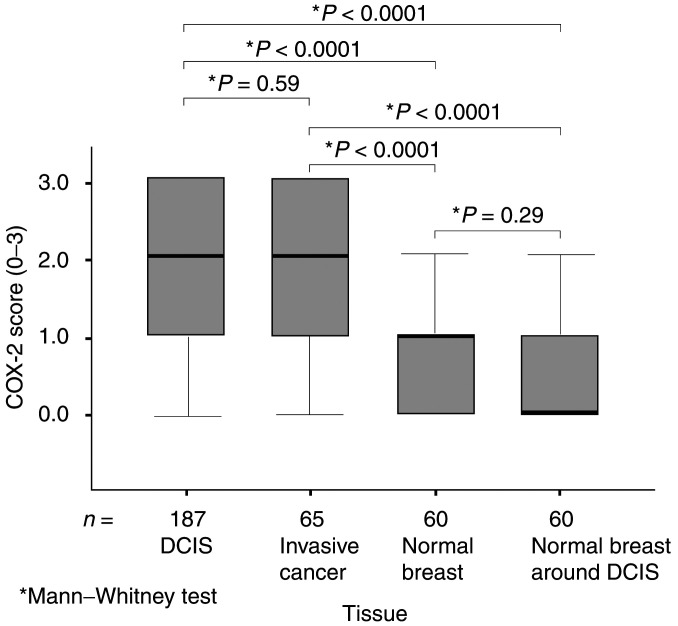
COX-2 expression in normal and neoplastic breast tissue. For each tissue, the thick black horizontal bars represent the median COX-2 score, the boxes represent the interquartile range and the T-bars the full range. The median COX-2 expression in neoplastic DCIS and invasive cancer epithelium are significantly greater than in normal breast ducts (*P*<0.0001), although there was no difference between DCIS and invasive cancer in median expression (*P*=0.59).

). In all cases, positive cellular immunostaining was cytoplasmic. Strong immunoreactivity was also observed in the smooth muscle of the tunica media of blood vessels and in the myoepithelial cells surrounding neoplastic breast ducts. No immunoreactivity was seen in stromal cells or in vasculature around normal breast ductules.

Cyclooxygenase type-2 expression in pure DCIS (*n*=187) and IBC (*n*=65) was significantly higher than in normal breast tissue (either from breast reduction tissue, *n*=60 or from ducts surrounding DCIS, *n*=60, *P*<0.0001, Mann–Whitney test). There was, however, no difference in the median COX-2 expression between *in situ* and invasive cancer (*P*=0.59), or between normal tissue from breast reduction samples and normal ducts around DCIS (*P*=0.29). The percentage of COX-2 positivity (a staining score ⩾2+) for DCIS, invasive cancer and normal breast from reduction and around DCIS was 67, 63, 23 and 22, respectively. Comparable COX-2 Immunoreactivity was confirmed in DCIS with a different COX-2 primary antibody from the Cayman Chemical Company (Ann Arbor, MI, USA, 160112) in 50 DCIS sections.

### C0X-2 expression in DCIS and other clinico-pathological parameters

In DCIS, there was no association between COX-2 expression and patient age above and below the age of 50 years (*P*=0.34, Table 2

**Table 2:** COX-2 expression and clinicopathological parameters in women with DCIS (*n*=187) and IBC (*n*=65)

**Parameter**	**Number**	**% COX-2 +ve**	***P*-value^a^**
**DCIS**			
*Age (years)*			
<50	35	62%	0.34
⩾50	152	69%	
*Nuclear grade*			
Low	27	44%	
Intermediate	64	64%	0.003
High	96	75%	
*ER status*			
Positive	111	58%	0.003
Negative	75	80%	
*Ki67 cell proliferation*			
<10%	93	55%	<0.0001
⩾10%	90	79%	
*HER-2 status*			
Positive	102	82%	<0.0001
Negative	85	48%	

**Invasive cancer**			
*Age (years)*			
<50	23	64%	0.67
⩾50	42	62%	
*Nuclear grade*			
I	17	53%	
II	17	58%	0.43
III	21	71%	
*ER status*			
Positive	40	50%	0.005
Negative	25	84%	
*Ki67 cell proliferation*			
<10%	29	50%	0.04
⩾10%	36	74%	
*HER-2 status*			
Positive	29	79%	0.014
Negative	36	50%	

**Figure 2:**
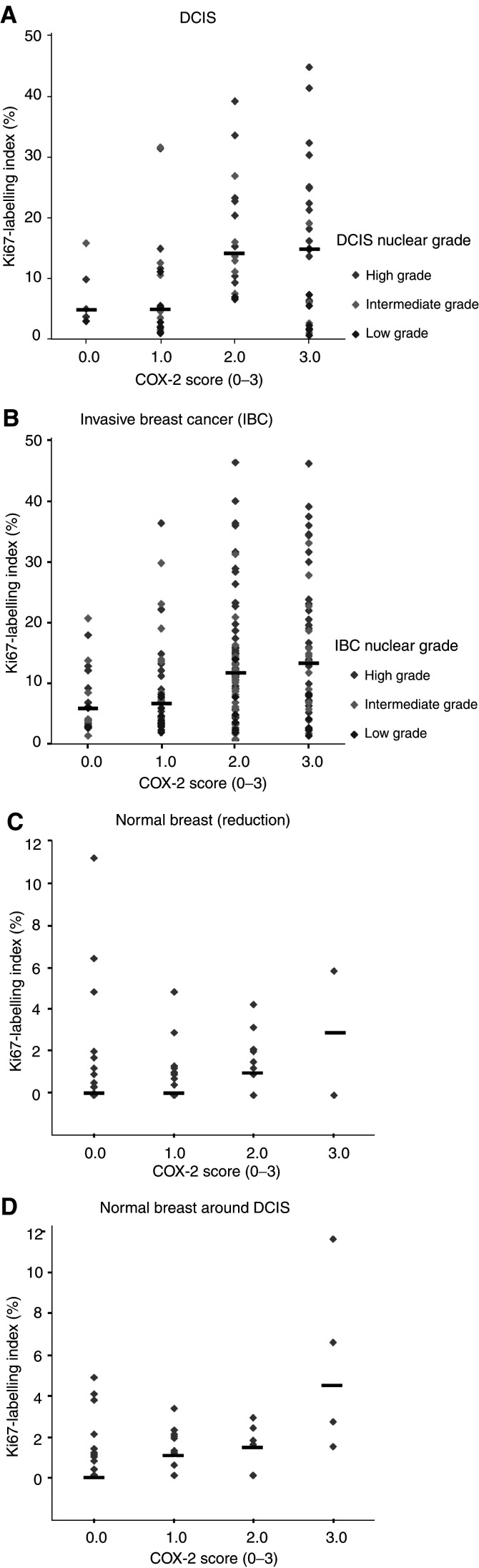
Association between COX-2 expression score and cell proliferation (Ki67-labelling index) for DCIS, IBC and normal breast. For DCIS and IBC, individual ki67 scores are separated by nuclear grade. The thick black horizontal lines represent the median Ki67 score for each COX-2 score (0–3) for each tissue. For each tissue type, there is a significant increase in Ki67 with increasing COX-2 score. In DCIS and IBC, the median Ki67 increases with increasing nuclear grade, although this is only significant for DCIS (*P*=0.003).

**Figure 3:**
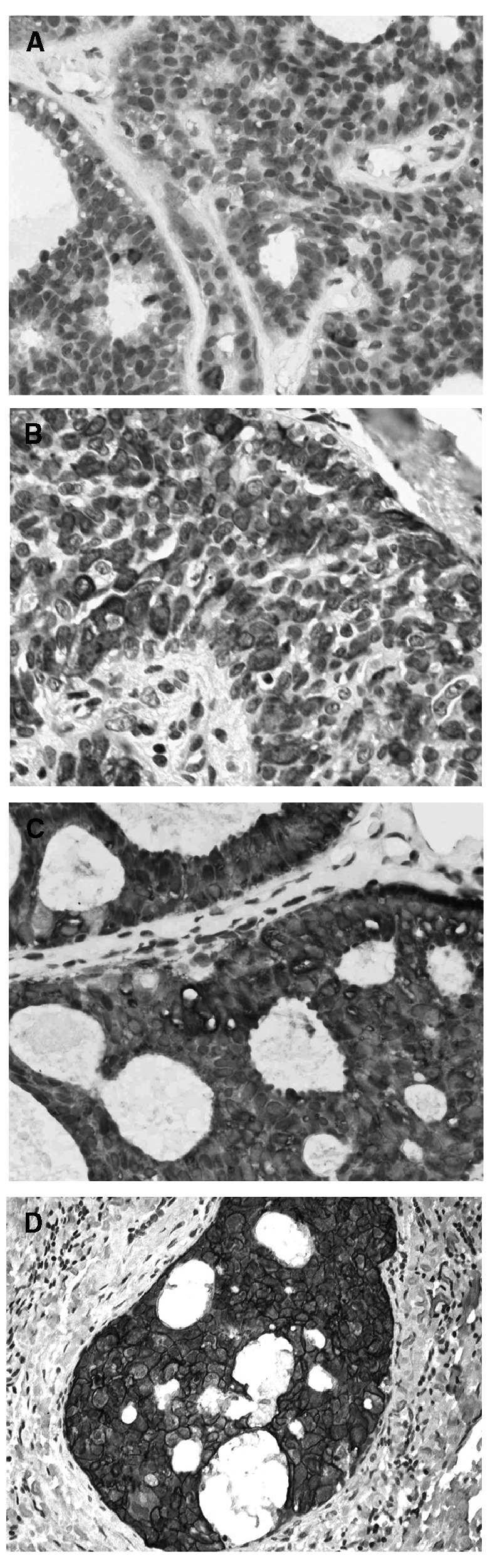
(**A**) DCIS showing minimal COX-2 expression with scanty brown cytoplasmic immunostaining (score 1+, classified as COX-2 negative), (**B**) moderate COX-2 staining with approximately 5060% of cells strongly stained (score 2+, classified as COX-2 positive), (**C**) High COX-2 expression in cribriform DCIS with 100% of cells intensely stained (score 3+, classified as COX-2 positive), (**D**) HER-2 staining in DCIS: note the strong membranous staining (score 3+, classified HER-2 positive).

Age above and below 50 years was used to separate pre- and postmenopausal patients crudely. Oestrogen receptor positivity was a tumour with ⩾5% nuclei expressing the ER receptor for both DCIS and IBC. A comparison of COX-2 positivity with Ki67 cell proliferation above and below 10% was used because the median DCIS cell proliferation was approximately 10%. HER-2 positivity was an immunostaining score of ⩾2+ (reference laboratory standard). Categorical variables were compared differences using the *χ*^2^ test. COX-2=cyclooxygenase type-2; DCIS=ductal carcinoma *in situ*; IBC=invasive breast cancer; ER=oestrogen receptor.

a *χ*^2^ test.

). Cyclooxygenase type-2 expression increased significantly with increasing nuclear grade (*P*=0.003), with the largest difference between intermediate (64% positivity) and high-grade (75% positivity) DCIS lesions compared to low-grade DCIS lesions (44% positivity, Table 2, Figure 2

). Similarly, there was a positive association of COX-2 immunoreactivity with cell proliferation in DCIS (*P*=0.004, Kruskal–Wallis test, Figure 2). The group of DCIS tumours with a ki67 cell proliferation of ⩾10% (*n*=90) was associated with 79% COX-2 positivity compared to 55% in the group with <10% (*n*=93) of cell expressing the Ki67 antigen (*P*<0.0001, *χ*^2^ test, Table 2). There was a significant association between ER negativity (40% of DCIS) and COX-2 positivity, with 80% of ER-negative DCIS showing COX-2 positivity compared to 58% of ER-positive DCIS tumours (*P*=0.003, *χ*^2^ test, Table 2). The overall HER-2 positivity rate for DCIS tumours selected for this series was 55%. Cyclooxygenase type-2 positivity was significantly higher in HER-2-positive DCIS tumours (82%) than HER-2-negative tumours (48%, *P*<0.0001, Table 2, Figure 3

).

### COX-2 expression in IBC and other clinco-pathological parameters

There was no significant association between COX-2 expression in IBC with patient age or with nuclear grade, although the percentage of COX-2 positivity was greater in high-grade compared to non-high-grade tumours (Table 2, Figure 2). Similar to DCIS, there was an association between COX-2 expression in IBC with a higher cell proliferation (Ki67, *P*=0.04, *χ*^2^ test), HER-2 positivity (*P*=0.014) and with ER negativity (*P*=0.005).

### COX-2 expression and cell proliferation in normal breast

Although the median cell proliferation was lower in normal breast compared to *in situ* and invasive cancer, there was a significant association between increasing cell proliferation and COX-2 expression in normal breast ductal epithelium in both reduction mammoplasty tissue (*P*=0.03, Mann–Whitney test) and from around DCIS (*P*=0.004, Figure 2).

## DISCUSSION

Cyclooxygenase type-2 is overexpressed along the continuum of carcinogenesis from preinvasive lesions to metastatic disease in tissues of both squamous and glandular origin (Eberhart *et al*, 1994; Kirschenbaum *et al*, 2000; Shirahama, 2000; Soslow *et al*, 2000; Morris *et al*, 2001; Costa *et al*, 2002). Cyclooxygenase type-2 overexpression in IBCs is associated with unfavourable prognostic indices (Ristimaki *et al*, 2002; Denkert *et al*, 2003; Spizzo *et al*, 2003). Evidence from both human and animal studies strongly suggests that cancer can be prevented by COX-2 inhibition.

This is the largest study to demonstrate elevated COX-2 expression in pure DCIS and has shown COX-2 overexpression in both *in situ* and IBC compared with normal breast. Since most IBC is believed to originate from DCIS (the two coexist in about 50% of cases), the inhibition of COX-2 represents a potential target for preventing breast cancer oncogenesis and as an adjuvant treatment following surgery to reduce local recurrence.

In this study, COX-2 expression was determined using immunohistochemistry on archival samples of breast tissue using scoring systems reported by others (Half *et al*, 2002; Ristimaki *et al*, 2002). Overall, COX-2 overexpression was demonstrated in 67% of DCIS and 63% of invasive tumours. For DCIS, this is consistent with the studies that have reported COX-2 in DCIS tumours (Soslow *et al*, 2000; Half *et al*, 2002; Shim *et al*, 2003; Watanabe *et al*, 2003).

The few published data with regard to COX-2 expression in normal breast tissue are conflicting. Using RT-PCR, Half *et al* (2002) reported a lower COX-2 mRNA level in normal breast tissue than in paired neoplastic tissue in eight of nine samples, while Watanabe *et al* (2003) found no COX-2 mRNA in normal breast tissue examined. Costa *et al* (2002) reported COX-2 protein expression in eight of 46 IBCs using Western immunoblotting, but found no COX-2 antigen in the adjacent normal tissue samples studied. This discrepancy can be partly explained by the paucity of ductal units in ‘normal breast’ (mostly stromal tissue in which COX-2 is not expressed) in comparison to neoplastic tissue.

By immunohistochemistry, COX-2 expression has been reported in normal breast ductules surrounding non-low-grade DCIS at levels equal (15%) or higher (85%) than in the index neoplastic lesion (Shim *et al*, 2003), although immuno-positivity decreases with distance. A similar finding was reported by Half and colleagues with expression in 81% of 48 samples, although they found staining to be focal and generally similar or decreased in intensity relative to adjacent neoplastic epithelia (Half *et al*, 2002). Watanabe *et al* (2003) reported a low level of COX-2 expression in 50% of normal epithelia surrounding DCIS, but in only 15% of normal epithelia surrounding invasive disease. This is confirms the findings of Soslow *et al* (2000), who reported expression in only three out of (17%) of normal breast tissue close to invasive cancer.

We demonstrated COX-2 immunoreactivity in 45% of normal breast adjacent to DCIS, consistent with the findings of Wanatabe *et al*, but we only scored this as COX-2 positive in 22% of cases. However, the normal ductules examined in the present study were on tissue sections taken from blocks of normal tissue harvested macroscopically close to the neoplastic tissue (not necessarily on ducts directly adjacent to the neoplastic lesion), which in reality may be up to 2 cm distal to the margin of the index neoplastic lesion. This may explain why our COX-2 positivity in adjacent normal breast is lower than that reported by others. We did not report expression in normal ducts surrounding invasive cancer. The present study is the first to report COX-2 expression in normal breast from reduction mammoplasty tissue. We found immunoreactivity in 58% of samples, but only scored this as COX-2 positive in 23%, which is not statistically different from the expression in normal ducts surrounding DCIS (*P*=0.29).

The COX-2 positivity (63%) rate in IBCs in this study is higher than in other studies (Soslow *et al*, 2000; Half *et al*, 2002; Ristimaki *et al*, 2002; Spizzo *et al*, 2003; Watanabe *et al*, 2003), which have reported expression in 36-56% of tumours. This disparity can be explained by the deliberate selection of HER-2-positive invasive tumours in this study (45%), chosen to compare COX-2 expression between HER-2-expressing and non-HER-2-expressing cancers. HER-2 is overexpressed in approximately 20-30% of IBCs (and therefore lower overall than in this study), and is an independent marker of poor prognostic disease (Tsutsui *et al*, 2002). An association between HER-2 and COX-2 expression in IBC has been reported in cell line (Vadlamudi *et al*, 1999; Subbaramaiah *et al*, 2002b), animal (Howe *et al*, 2002) and in human immunohistochemical studies (Ristimaki *et al*, 2002). Ristimaki *et al* (2002) found a higher COX-2 expression in HER-2-expressing breast cancer; the present study confirmed this association. Furthermore, dual drug blockade of COX-2 and HER-2 in cancer cell lines expressing both oncogenes has been shown to reduce cell growth more effectively than the inhibition by one of the agents alone (Mann *et al*, 2001). These studies suggest that signalling through the HER-2 receptor may have a role in modulating the upregulation of COX-2 in IBC.

Since HER-2 overexpression is reported in up to 80% of DCIS tumours and is associated with high-grade phenotype (Allred *et al*, 1993; Bobrow *et al*, 1995; Suo *et al*, 2001), we investigated the association of COX-2 and HER-2 expression in DCIS. The overall HER-2 positivity rate for DCIS in this study was only 55%, reflecting the deliberate inclusion of a higher proportion of non-high-grade DCIS tumours in this study (associated with a lower HER-2 positivity) to investigate COX-2 expression across nuclear grades. Consistent with the findings in invasive tumours, we found that COX-2 positivity was significantly higher in HER-2-positive DCIS (82%) than in HER-2-negative DCIS (48%). Importantly, this finding in *in situ* breast cancer confirms the association of the HER-2 receptor with COX-2 overexpression reported in breast cancer cell line studies (Vadlamudi *et al*, 1999; Subbaramaiah *et al*, 2002a) (and by immunohistochemistry; Ristimaki *et al*, 2002), which suggest that signalling through HER-2/Ras/Map kinase pathway may play a role in upregulating COX-2 in neoplasia and this may occur at the preinvasive stage of breast cancer carcinogenesis. This could explain why COX-2 expression is higher in DCIS than IBC.

Inhibition of the COX-2 enzyme and attenuation of the consequential carcinogenic effects of overexpression with increased prostaglandin production (inhibition of apoptosis, stimulation of neo-angiogenesis, upregulation of intra-tumoral CYP19 aromatase; Davies *et al*, 2002) at this stage has the potential to prevent progression to invasion.

In DCIS tumours, we also found COX-2 expression to be positively associated with higher cellular proliferation rates, higher nuclear grade and with ER negativity. These findings, together with the association of COX-2 with HER-2 expression, are consistent with those reported in IBC (Ristimaki *et al*, 2002). This is important since these factors are surrogate markers of an aggressive DCIS phenotype and link nonoestrogen growth factor signalling pathways with COX-2 overexpression. The association of COX-2 expression with nuclear grade in DCIS confirms the findings of Shim *et al* (2003). Since nuclear grade is an independent marker of DCIS local recurrence (Silverstein *et al*, 1996; Boland *et al*, 2003a), further study is warranted to determine whether COX-2 is independent of grade with regard to the risk local DCIS recurrence following breast conservation.

These findings may have important therapeutic and cancer chemo-preventative implications. Since signalling through HER-2 pathways is believed to be involved in driving cell proliferation in ER-negative IBC and to resist anti-oestrogen therapy in ER-expressing/HER-2-positive cancers, the association of COX-2 with HER-2 expression in DCIS suggests that the carcinogenic sequalae of COX-2 overexpression originate at the preinvasive stage in breast carcinogenesis.

There is a clinical need to determine whether women with HER-2-positive/COX-2-positive DCIS represent a high-risk patient population for disease progression or local recurrence; this cohort especially may benefit form COX-2 inhibitor therapy. Phase II clinical trials are presently in progress to determine the efficacy of COX-2 inhibition combined with Herceptin in HER-expressing metastatic breast cancer.

The targeting of nonhormonal pathways will be necessary to prevent both ER-positive and ER-negative breast cancer. Since COX-2 inhibition is a relatively safe therapeutic option, we believe that prospective clinical trials are warranted to determine the clinical benefit of long-term COX-2 inhibition in preventing breast cancer and as an adjuvant therapy after DCIS treatment.
